# Management of Patients Treated with Direct Oral Anticoagulants in Clinical Practice and Challenging Scenarios

**DOI:** 10.3390/jcm12185955

**Published:** 2023-09-13

**Authors:** Fabiana Lucà, Fabrizio Oliva, Maurizio Giuseppe Abrignani, Stefania Angela Di Fusco, Iris Parrini, Maria Laura Canale, Simona Giubilato, Stefano Cornara, Martina Nesti, Carmelo Massimiliano Rao, Andrea Pozzi, Giulio Binaghi, Alessandro Maloberti, Roberto Ceravolo, Irma Bisceglia, Roberta Rossini, Pier Luigi Temporelli, Antonio Francesco Amico, Raimondo Calvanese, Sandro Gelsomino, Carmine Riccio, Massimo Grimaldi, Furio Colivicchi, Michele Massimo Gulizia

**Affiliations:** 1 Cardiology Department, Grande Ospedale Metropolitano, AO Bianchi Melacrino Morelli, 89129 Reggio Calabria, Italy; 2 Cardiology Department De Gasperis Cardio Center, Niguarda Hospital, 20162 Milan, Italy; 3 Operative Unit of Cardiology, P. Borsellino Hospital, 91025 Marsala, Italy; 4 Clinical and Rehabilitation Cardiology Department, San Filippo Neri Hospital, ASL Roma 1, 00135 Roma, Italy; 5 Cardiology Department, Ospedale Mauriziano, 10128 Turin, Italy; 6 Cardiology Department, Nuovo Ospedale Versilia Lido di Camaiore Lucca, 55049 Camaiore, Italy; 7 Cardiology Department, Cannizzaro Hospital, 95126 Catania, Italy; 8 Arrhytmia Unit, Division of Cardiology, Ospedale San Paolo, Azienda Sanitaria Locale 2, 17100 Savona, Italy; 9 Fondazione Toscana G. Monasterio, 56124 Pisa, Italy; 10 Cardiology Division Valduce Hospital, 22100 Como, Italy; 11 Department of Cardiology, Azienda Ospedaliera Brotzu, 09047 Cagliari, Italy; 12 Cardiology Unit, Giovanni Paolo II Hospital, 88046 Lamezia, Italy; 13 Integrated Cardiology Services, Department of Cardio-Thoracic-Vascular, Azienda Ospedaliera San Camillo Forlanini, 00152 Rome, Italy; 14 Cardiology Unit, Ospedale Santa Croce e Carle, 12100 Cuneo, Italy; roberta.rossini2@gmail.com; 15 Division of Cardiac Rehabilitation, Istituti Clinici Scientifici Maugeri, IRCCS, 28010 Gattico-Veruno, Italy; 16 Cardiovascular Prevention and Ortokinesis Clinics of Lecce, 73043 Lecce, Italy; 17 Cardiology Unit, Ospedale del Mare, 80147 Napoli, Italy; 18 Cardiovascular Research Institute, Maastricht University, 6211 LK Maastricht, The Netherlands; 19 Cardiovascular Department, Sant’Anna e San Sebastiano Hospital, 81100 Caserta, Italy; 20 Department of Cardiology, General Regional Hospital “F. Miulli”, 70021 Bari, Italy; 21 Cardiology Department, Garibaldi Nesima Hospital, 95122 Catania, Italy

**Keywords:** direct oral anticoagulants (DOACs), atrial fibrillation (AF), vitamin K antagonists (VKAs), adherence, cancer, malignancy, chronic kidney disease (CKD), chronic liver disease (CLD), drug–drug interactions (DDIs), triple antithrombotic therapy (TAT), dual antiplatelet therapy (DAPT), pacemaker, implantable cardioverter-defibrillator (ICD) implantation, Catheter Ablation of Atrial Fibrillation (CAAF), non-cardiac surgery, elderly, frailty, obesity, Under-Weight Patients, Over-Weight Patients

## Abstract

It is well established that direct oral anticoagulants (DOACs) are the cornerstone of anticoagulant strategy in atrial fibrillation (AF) and venous thromboembolism (VTE) and should be preferred over vitamin K antagonists (VKAs) since they are superior or non-inferior to VKAs in reducing thromboembolic risk and are associated with a lower risk of intracranial hemorrhage (IH). In addition, many factors, such as fewer pharmacokinetic interactions and less need for monitoring, contribute to the favor of this therapeutic strategy. Although DOACs represent a more suitable option, several issues should be considered in clinical practice, including drug–drug interactions (DDIs), switching to other antithrombotic therapies, preprocedural and postprocedural periods, and the use in patients with chronic renal and liver failure and in those with cancer. Furthermore, adherence to DOACs appears to remain suboptimal. This narrative review aims to provide a practical guide for DOAC prescription and address challenging scenarios.

## 1. Introduction

Direct oral anticoagulants (DOACs) are currently considered the anticoagulation strategy of choice for thromboembolic prevention in several cardiovascular conditions, such as atrial fibrillation (AF) and venous thromboembolism (VTE) [1,2], and should be preferred over other treatments in eligible patients [3,4].

Indeed, DOACs, classified as direct oral factor Xa inhibitors (apixaban, rivaroxaban, and edoxaban) and direct thrombin inhibitors (dabigatran), have been demonstrated to be superior or non-inferior to vitamin K antagonists (VKAs) in lowering the risk of thromboembolic events without increasing bleeding risk [2,5]. Moreover, many advantages have been reported, including the fact that they do not need coagulation parameters, have fewer nutrient and drug–drug interactions (DDIs), and present fewer other pharmacokinetic and pharmacodynamic issues, such as genetic polymorphisms, which may affect VKAs’ activity [2].

However, several aspects of DOAC management remain challenging, including DDIs, switching to other therapies, periprocedural and postprocedural management strategies, and the use in patients with chronic renal and liver failure and in those with cancer [2]. Moreover, according to real-world data, adherence to DOACs seems to be scarce, remaining at approximately 40% of DOAC patients [6].

The aim of this review is to provide physicians with a practical guide for DOAC prescribing, providing pragmatic suggestions for the most controversial scenarios.

## 2. Methods

The literature search was performed using PubMed, Web of Science, and Google Scholar Databases. The PubMed Database was selected as the main database to perform this search. The PubMed search items used were the following: (“direct oral anticoagulants (DOACs)” [Mesh] OR “novel oral anticoagulants (NOACs)”) AND (“atrial fibrillation” [Mesh] OR “atrial fibrillation”) AND (“Adherence”) and (“Cancer” OR “Malignancy”), AND (“Chronic Kidney Disease (CKD)” AND (“Chronic Liver Disease” (CLD)) AND (“Drug-Drug Interactions” (DDIs)) AND (“Triple Antithrombotic Therapy” (TAT)) AND (“Dual Antiplatelet Therapy” (DAPT)) AND (“Pacemaker”) AND (“Implantable cardiovert-er-defibrillator (ICD) implantation”) AND (“Catheter Ablation of Atrial Fibrillation” (CAAF)) AND (“Non-Cardiac Surgery”) AND (“Elderly”) AND (“Frailty”) AND (“Obesity”) AND (“Under-Weight Patients”) AND (“Over-Weight Patients”). Only articles written in English were examined. The search strategy was decided by two authors (FL and MGA), and a third author (SADF) approved the decisions.

## 3. Drug–Drug Interactions (DDIs) of DOACs and Polypharmacy

Although fewer DDIs have been ascribed to DOAC than expected using warfarin [7], the concomitant administration of agents that affect DOAC plasma concentrations may increase or reduce DOAC effects, potentially increasing bleeding or ischemic risk, respectively.

The principal interactions of DOACs that need to be considered concern drugs that influence renal and hepatic clearance and drugs that affect hemostasis [8]. The interactions with the efflux transporter P-glycoprotein (P-gp) and CYP3A4-type cytochrome P450 (CYP3A4/5) must also be carefully taken into account [7].

From the perspective of DDI, medications can be categorized as inducers or inhibitors of one or more of these enzymes or transport proteins. In cases of inhibition, there arises direct competition between medications, leading to heightened serum concentrations of either one or both agents. Conversely, induction results in diminished serum concentrations, potentially compromising the effectiveness of a medication [9,10].

With regard to CYP450 enzymes, those most commonly involved in DDI are CYP1A2, CYP2C9, CYP2C19, CYP2D6, CYP3A4, and CYP3A5 [9,11].

It has been shown that hepatic clearance of both rivaroxaban and apixaban is CYP3A4 type cytochrome P450-dependent [12,13], considering the fact that CYP3A4 contributes to approximately 50% and 20–25% of their respective metabolic pathways [10]. This differs from dabigatran, which does not act as a substrate, inhibitor, or inducer of Cytochrome P450 enzymes, and edoxaban, where less than 4% of its metabolism is facilitated by the CYP3A4 enzyme [9,10].

P-gp belongs to the multidrug resistance protein 1 (MDR1) family, which is encoded by the ATP-binding cassette subfamily B (ABCB1) gene [9].

Additionally, P-gp operates as an efflux pump, playing a pivotal role in mitigating tissue exposure to compounds with potentially deleterious effects, thereby facilitating their efflux and removal [10,11].

P-GP takes part in the renal excretion of DOACs [8]. Thus, the P-GP competitive inhibition will increase DOAC plasma values [14].

P-gp acts as a substrate for apixaban, dabigatran, and rivaroxaban, while it does not exhibit any significant activity on edoxaban [9].

However, also other transport proteins and enzymes have been hypothesized to be involved in DDIs, such as the influx transporter, the organic anion-transporter polyprotein (OATP), the efflux/influx organic transporter (OCT), and the efflux transporter breast cancer resistance protein (BCRP), expressed in the intestinal, hepatic, and biliary sites [9]. This fact may represent a problem because AF or VTE patients are commonly politreated. By and large, polymedication has been correlated with increased mortality and bleeding [15,16,17].

The effects of DDIs have been extensively described in the ESC practical guide on DOACs [18]. Strong CYP3A4/5 and P-GP/CYP3A4/5 inhibitors can significantly increase DOAC plasma levels and increase the risk of bleeding [19,20]. If strong CYP3A4/5 inhibitors need to be used, it is preferable to use dabigatran, edoxaban, or VKAs rather than apixaban and rivaroxaban, considering that CYP3A4/5 affects their metabolism more. Of note, only for edoxaban, a dose reduction is recommended in patients concomitantly taking potent P-GP inhibitors, including dronedarone, verapamil, and quinidine, according to the ENGAGE study [21]. Since all DOACs are substrates for PGP, strong PGP/CYP 3A4/5 inhibitors expose patients to increased bleeding risk with all DOACs [7]. Antifungals, macrolides, and antiretroviral protease inhibitors are potent inhibitors of PGP that interact with DOACs [7].

DOACs may be cautiously used concomitantly with moderate and weak inhibitors of PGP or CYP 3A4/5, providing that other bleeding risk factors such as renal dysfunction (clearance < 50 mL/min), weight < 60 kg, or advanced age (>80 years) did not coexist. In addition, for rivaroxaban, the presence of a mild or moderate hepatic failure (Child-Pugh A or B) represents a condition that requires careful management if moderate-weak inhibitors of PGP or CYP 3A4/5 have to be contemporarily used. Moreover, when a moderate- weak PGP or CYP 3A4/5 inhibitor must be associated with DOACs, if more than two bleeding risk factors occur, or if a severe renal dysfunction coexists (clearance < 30 mL/min), it should be reasonable to use VKAs as a first-line treatment or, if it is possible, to choose a non-interacting drug.

The assessment of DOAC plasmatic levels and the use of “off-label” reduced doses are not supported by consistent evidence, so this strategy is not suggested for most patients. In particular, the determination of DOAC plasma concentrations should be limited to rare cases when potentially significant interactions occur or for specific conditions as an option only for experienced centers.

On the other hand, moderate-strong PGP/CYP3A4/5 inducers’ use should be avoided in all patients taking DOACs as they determine a significant reduction in DOAC concentration. Therefore, VKAs may be considered the best option in these cases.

For example, DOACs administration is contraindicated in patients treated with rifampicin, a potent inducer of PGP. Rivaroxaban and apixaban labels advise avoiding the use of carbamazepine, phenytoin, phenobarbital, concomitant P-GP, and CYP3A4 inducers.

A list of the most common drugs, moderate to strong Pgp/CYP3A4/5 inhibitors, and inducers is shown in Table 1.

Although DOACs are recognized for their predictable pharmacokinetics and pharmacodynamics, recent observations have drawn attention to a wider spectrum of inter-individual variability encompassing both plasma concentrations and drug responses. This variability can be influenced by several factors, including age, race, gender, smoking, and dietary patterns. Moreover, the presence of common genetic variations or interactions between drugs may also contribute to the manifestation of these differences [22].

The exploration of pharmacogenomics in relation to DOACs constitutes a relatively nascent field of investigation. However, it should place more emphasis on personalized medication management and pharmacogenomic testing to optimize DOAC prescribing in patients with potential interactions. Moreover, it is advisable to gain insight into the contribution of pharmacogenomics to the interpatient diversity observed in DOAC responses. Indeed, the variability exhibited in DOAC responses can be partially ascribed to genetic variants within specific gene loci as well as drug–drug interactions [22].

Remarkably, genetic variants within carboxylesterase 1 (CES1) and ABCB1 with multiple single nucleotide polymorphisms (SNPs) are among the most extensively documented, contributing to notable modifications in the peak and trough levels of dabigatran, yielding discernible clinical ramifications. Similarly, ABCB1 SNPs exert an influence on the modulation of plasma drug concentrations of rivaroxaban and apixaban. Conversely, investigations involving genetic variants such as factor Xa, ABCB1, Solute Carrier Organic Anion Transporter Family Member 1B1 (SLCOB1), CYP2C9, and Vitamin K epOxide Reductase Complex (VKORC1) subunit 1 did not uncover any substantial associations with the plasma drug levels of edoxaban [22].

## 4. How to Switch between Different Anticoagulants

Patients on anticoagulation therapy may need a switch from DOACs to VKAs or vice versa for several reasons [23]. In these cases, it is of paramount importance to consider the different pharmacokinetic and pharmacodynamics proprieties among different anticoagulants, ensuring the continuation of therapy and reducing the bleeding risk.

VKAs to DOACs. According to the latest international recommendations, an INR < 2 allows the immediate initiation of DOACs, whereas, in the presence of INR 2.0–2.5, DOACs could be administrated promptly or postponed to the next day [24]. If the patient has an INR value above 2.5, it is advisable to take into account the time needed for obtaining an INR lower than the threshold value, considering a half time of 8–24 h for acenocoumarol, 36–48 h for warfarin, and six days for phenprocoumon [24]. At this time, a second INR measurement is recommended before DOAC administration. When DOACs are started, no further INR measurements are needed [24].

DOACs to VKAs. Due to the slow time to achieve the therapeutic range of VKAs, from 5–10 days, a simultaneous administration of DOACs and VKAs is recommended until INR reaches an appropriate therapeutic value [24]. No loading dose is recommended for VKAs, but for phenprocoumon. Notably, since DOACs may affect INR, this should be closely monitored for at least one month after DOACs withdraw, until stable INR values are achieved. Particularly, 2–3 days after stopping DOACs, a new measurement is strongly suggested to ensure adequate anticoagulation.

If concurrent DOAC administration during VKA treatment initiation is considered inappropriate (i.e., severe acute renal impairment with secondary increased DOAC blood levels), switching from DOACs to Low-Molecular-Weight Heparin (LMWH) with concomitant VKAs administration may be a suitable option, chiefly in high thromboembolic risk patients [24].

DOACs to DOACs. The alternative DOACs should be administrated at the time the next dose of the initial DOACs is expected. It has been suggested that a longer interval is seen in the presence of situations in which plasma concentrations are expected to be higher than the therapeutic concentration (i.e., renal impairment). However, no further and more detailed recommendations based on time administration have been provided.

DOACs to parenteral or subcutaneous anticoagulation. Parenteral or subcutaneous anticoagulation treatment should be started when the next DOAC dose is due [24]. However, if ST-elevation myocardial infarction (STEMI) occurs, enoxaparin or unfractionated heparin (UFH) should be used regardless of the time of the last dose of DOACs [24].

Parenteral anticoagulant to DOACs. Due to a very short half-life of intravenous UFH (2 h), DOACs can be started 4 h after intravenous UFH discontinuation [24]. Conversely, in the presence of LMWH treatment, DOACs should be administrated when the next LMWH dose is due, with particular attention if a renal dysfunction coexists wherein LMWH catabolism may be longer.

DOACs in patients with Chronic kidney disease (CKD)

Chronic kidney disease (CKD) and AF may increase thromboembolic risk through several mechanisms [25]. Moreover, the bleeding risk has been shown to rise in CKD. The risk of bleeding is particularly high in the presence of GFR < 30 mL/m, CKD-related anemia, polytherapy, and invasive procedures.

The gradual development of renal impairment has been estimated to increase the risk of cerebral hemorrhage up to 10-fold in dialysis patients [26].

In addition, GI bleeding has been reported to increase with worsening renal function [27].

For this reason, managing AF patients with concomitant CKD is particularly challenging.

Patient risk stratification should be performed using the CHA_2_DS_2_VASc and HAS-BLED scores. Before starting oral anticoagulant therapy, all measures to lower bleeding risk must be implemented. All DOACs are eliminated by the kidney; renal excretion of 80%, 50%, 35%, and 27% has been observed for dabigatran edoxaban, rivaroxaban, and apixaban, respectively.

Therefore, before administering DOACs, an evaluation of renal function is required [28].

Any condition that may worsen renal function (infection, acute heart failure, potentially nephrotoxic drugs, etc.) requires additional checks. Obviously, DOACs should be suspended for patients with acute renal failure [29].

The dosage to use in patients with mild-moderate CKD, defined as a glomerular filtration rate (GFR) between 30 and 50 mL/min, depends on each DOAC’s kidney elimination. In these patients, dabigatran, 110/150 mg can be used according to the patient’s clinical characteristics, apixaban 2.5/5 mg should be used following specific parameters extrapolated from phase 3 clinical trials, and the dosage of rivaroxaban must be reduced to 15 mg if creatinine serum levels are >1.5 mg/dL and edoxaban needs to be reduced at 30 mg when GFR is between 15 and 49 mL/min [18]. In the US and Europe, the use of low doses of rivaroxaban, apixaban, and edoxaban (but not dabigatran) has been approved with a CrCl of 15–29 mL/min [18], although data on outcomes are poor.

According to the guidelines for GFR < 15 mL/min, DOACs are contraindicated considering the fact that phase 3 clinical trials did not include dialytic and advanced renal dysfunction individuals [18].

Moreover, limited studies on apixaban and rivaroxaban in patients with end-stage renal disease (ESRD) and/or receiving hemodialysis hypothesize safety and efficacy findings in this complex category [30,31,32].

However, whether patients with severe renal impairment may benefit from DOACs and, furthermore, which drug should be used in this case, has yet to be substantially confirmed. When a patient is on dialysis or has a GFR < 15 mL/min, guidelines suggest an individualized approach that includes a multidisciplinary team discussion and patient engagement with a shared decision approach after being informed of the off-label use of drugs [29,32,33].

Another point regarding DOACs and CKD that deserves to be mentioned is the risk of CKD progression determined by anticoagulant treatments. Indeed, anticoagulant-related nephropathy is a rare disease determined by renovascular calcification and intrarenal hemorrhages. This condition can affect patients using both warfarin and DOACs, presenting as an acute kidney injury of a progressive CKD cell cast in renal tubules [33,34].

In conclusion, in patients with CHD, the appropriate dosages of DOACs based on patient GFR are considered to be crucial (Table 2).

## 5. DOACs in Patients with Advanced Chronic Liver Disease (CLD)

**Figure 1:**
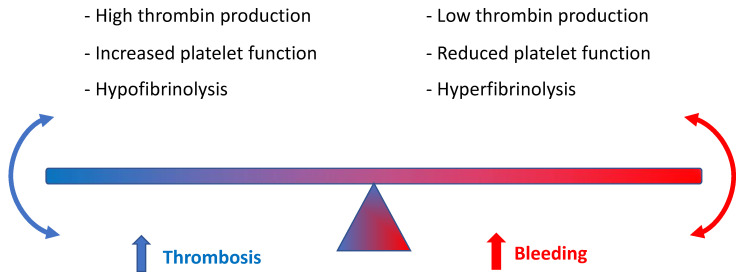
Role of hepatic function in balancing homeostasis and anti-thrombotic function.

Normal hepatic function is of paramount importance to balance homeostasis and anti-thrombotic function [35,36,37] (Figure 1). In this regard, people with advanced chronic liver disease (CLD) run a greater risk of both thrombosis and bleeding risk, which is related to thrombocytopenia (secondary to reduced thrombopoietin production) and reduced synthesis of fibrinogen or other coagulation factors such as II, V, VII, IX, X, XI, and XII. Conversely, low levels of protein C, antithrombin, and plasminogen, and, at the same time, hypercoagulability due to enhanced von Willebrand factor activity imply increased thrombotic risk. Of note, advanced CLD is commonly associated with prolonged prothrombin time [38,39].

The aforementioned characteristics represent a great challenge in managing OAC therapy when AF or VTE are associated with CLD. Moreover, data concerning DOACs in patients with CLD are poor since they were scarcely represented in DOAC trials [21,40,41,42].

However, the effect and drug concentration of DOACs may be affected by the presence of CLD considering the fact that hepatic metabolism of 75%, 65%, and 50% has been reported for apixaban, rivaroxaban, and edoxaban, respectively [18]. Conversely, dabigatran etexilate is a prodrug and its metabolism is not affected by hepatic function.

The European Medicines Agency (EMA) and the Food and Drug Administration (FDA) consider the Child–Pugh classification to guide DOACs use in patients with CLD. The Child–Pugh classification is a score contemplating clinical (ascites and encephalopathy) and serum values such as albumin, bilirubin, prothrombin time, or INR parameters to evaluate the prognosis of patients with CLD. The Child–Pugh classification classifies patients with mild (class A), moderate (class B), and severe (class C) liver impairment [18].

According to the FDA label recommendation, apixaban does not need any dose adjustment in patients with Child–Pugh A. As for Child–Pugh B patients, its use has been suggested with caution in consideration of scarce data, even though dose adjustment is not needed. Conversely, in Child–Pugh C patients, apixaban use is not recommended [18]. Notably, liver function should be assessed before administering apixaban in patients with CLD due to the fact that if a concomitant coagulation disorder and clinically relevant bleeding risk coexist, its use should be avoided [18].

As previously reported, two-thirds of rivaroxaban have hepatic metabolism. According to the pharmacokinetic and pharmacodynamic properties, rivaroxaban should be avoided in Child–Pugh B and C classes or in the presence of a concomitant coagulopathy.

Due to the high hepatic metabolism of edoxaban, any dose adjustment in Child–Pugh A patients is required. In contrast, this Xa inhibitor is not recommended in Child–Pugh B and C adults. Conversely, according to the EMA recommendation, edoxaban does not need any dose adjustment in Child–Pugh A and B patients, whereas in Child–Pugh C patients, it is not recommended. Additionally, according to EMA, these Xa inhibitors should not be used in CLD patients with coagulation disorders and in those at high bleeding risk [18].

Dabigatran etexilate is the sole prodrug among DOACs, and due to a reduced metabolized fraction in the liver, hepatic impairment has less influence on its metabolism. According to DOACs’ pharmacokinetics and pharmacodynamics, patients with mild or moderate CLD should not have a dose adjustment, whereas, in the Child–Pugh C population, dabigatran is not recommended [18]. Conversely, the use of dabigatran should be avoided in patients with a 2-fold increased upper limit of hepatic enzyme regardless of hepatic impairment, and the oral thrombin inhibitor is not recommended in patients with advanced CLD.

As previously reported, patients with CLD run a higher bleeding risk because of the reduced production of pro-thrombotic agents. [18].

Differently from patients with renal impairment, there is a paucity of information regarding DOAC use in patients suffering from liver disorders.

Indeed, the CLD population has been largely excluded from pivotal randomized controlled trials investigating antithrombotic medications. This has led to a poor agreement regarding the safety, effectiveness, and monitoring protocols associated with anticoagulant and antiplatelet therapies in patients with CLD. As a consequence, the optimal strategy for DOAC in the CLD patient cohort remains an area of uncertainty.

Importantly, to date, no prospective clinical trial has examined the safety and efficacy of DOACs in reducing thrombotic events in patients with CLD, and available data have been obtained from pharmacokinetic investigations, case reports, and limited-scale observational research [43,44,45,46].

Furthermore, managing CLD patients in terms of anticoagulation therapy is particularly challenging in high-risk contexts such as acute coronary syndrome (ACS) and urgent percutaneous revascularization (PCI). Indeed, altered hemostatic mechanisms have been associated with liver impairment so OAC use combined with one or more antiplatelet therapies should be restricted to the shortest period in this high-risk population.

Considering the growing incidence of AF and CAD in this subset of patients and the expanding range of therapeutic options, data were extrapolated from real-world analyses [43].

DOAC-treated patients with advanced CLD and bleeding episodes should be considered as those without hepatic dysfunction. Thrombin time, diluted thrombin time, and ecarin clot time are the coagulation tests used in patients on dabigatran. In contrast, a calibrated chromogenic anti-Xa assay is commonly used in patients treated with Xa inhibitors [47,48,49]. These tests assess the blood concentration of DOACs. If the concentration exceeds 30 ng/mL, reversal agents such as Idaracuzimab and Andexanet alfa for dabigatran and Xa inhibitor, respectively, should be considered [18,50,51].

## 6. DOACs Management in the Preoperative and Postoperative Setting

### Anticoagulant and Antiplatelet Combined Therapy

**Figure 2:**
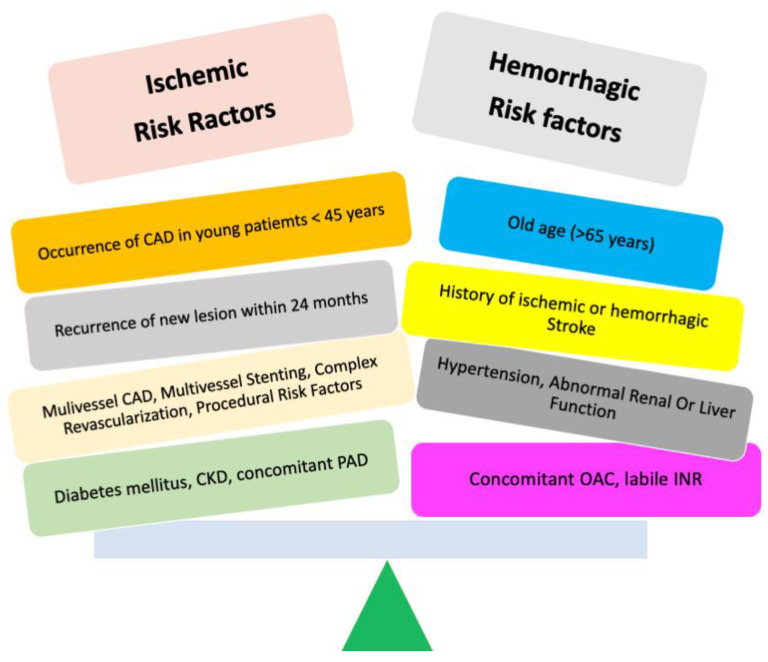
Balance on ischemic and bleeding risk factors.

**Figure 3:**
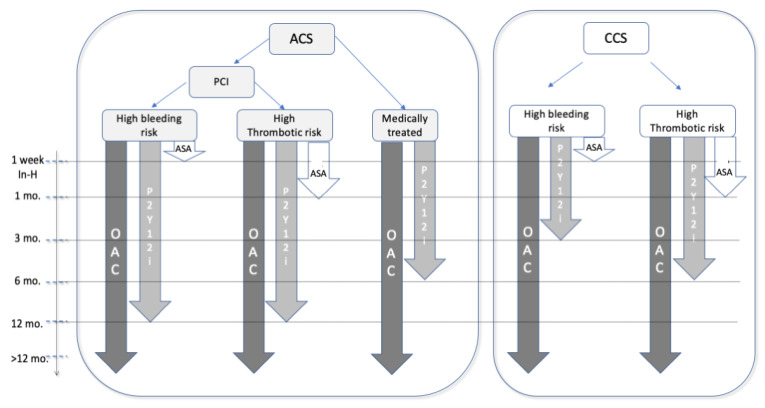
Management of anticoagulant and antiplatelet strategies in different coronary setting Abb: ACS: acute coronary syndrome; PCI: Percutaneous Coronary Intervention; CCS: chronic coronary syndrome: OAC: oral anticoagulant; P2Y12i: P2Y12 inhibitor; ASA: aspirin; mo.: month.

Given the frequent coexistence of coronary artery disease (CAD) and AF, an essential issue of polypharmacy is represented by the co-administration of DOACs and antiplatelet therapy (APT) [8] due to the concomitance of ischemic, thromboembolic, and bleeding risk (Figure 2). Indeed, combined antithrombotic therapy (AT) with anticoagulants and APT implies an increased risk of bleeding [52]. For this reason, the latest guidelines [53] recommend minimizing the duration of combined AT. In most cases, triple antithrombotic therapy (TAT) consisting of an oral anticoagulant and dual antiplatelet therapy (DAPT), usually aspirin and clopidogrel, should be reduced to one week after ACS and/or PCI. Instead, in cases of high ischemic risk and no high bleeding risk, TAT should be prolonged for up to one month (Figure 3). In any case, DOACs in TAT or dual antithrombotic therapy (DAT) should be favored over VKAs due to their more favorable risk/benefit profile [4,52,53].

Considering the clinical relevance of possible DDIs, it is crucial that in each DOAC treatment follow-up visit, new co-medications are investigated and optimal DOACs and correct dosages are re-checked.

## 7. Elective and Urgent PCI

A 40% concomitant CAD prevalence has been reported in AF patients. Importantly, most of them need a revascularization strategy [54]. Moreover, it has been shown that up to 15% of patients undergoing elective or urgent percutaneous coronary intervention (PCI) also have AF, requiring an oral anticoagulant (OAC) for AF [54,55,56,57].

Notably, AT is required in patients undergoing PCI, representing the most common revascularization modality [54,56,57]. Therefore, AF patients undergoing PCI require both OAC for AF-related embolic event prophylaxis and DAPT with aspirin and a P2Y12 inhibitor to face stent thrombosis and atherosclerotic progression; consequently, their management becomes challenging [3,14,58,59,60]. Indeed, the use of TAT combining OAC with DAPT substantially raises the risk of bleeding [57], and, notably, bleeding occurrence after PCI may result in a worse outcome [14,61,62].

Therefore, concomitant ischemic and bleeding risk should be carefully evaluated and balanced in terms of the duration, type, and doses of combined antithrombotic therapy [63], taking into account that a significantly lower major bleeding (MB) risk has been reported using DAPT compared to TAT.

Nevertheless, a short period of TAT (≤one week) should be prescribed in AF patients who recently experienced a PCI, especially if an increased risk of ischemic events has been estimated [64,65].

However, each patient should be managed with a personalized antithrombotic/antiplatelet approach.

According to the specific patient’s bleeding and thrombotic risk, the anatomical and procedural profile is important in order to accurately characterize patients who are more likely to benefit from a more extended TAT regimen and avoid a prolonged TAT in those with a greater hemorrhagic risk. Moreover, the DOAC dosage should be evaluated, taking into account clinical features such as frailty, renal function, and the occurrence of dose reduction.

Notably, a reduced dose of DOACs should be used in association with APT if a high bleeding risk coexists [4].

Antithrombotic strategies in AF patients undergoing PCI have been investigated in dedicated RCTs and meta-analyses [66,67,68,69,70] in order to demonstrate the superiority or non-inferiority of DAT in comparison to TAT in terms of bleeding events [71]. However, only two of these trials exhibited a decrease in significant bleeding events in the DAT approach [67,68]. Additionally, the increased risk of ischemic events was not thoroughly assessed. Indeed, although there was no statistically significant rise in thrombotic complications between DAT and TAT groups, none of the trials were statistically powered to definitively rule out such a distinction [71].

In the ENTRUST AF PCI, it was observed that the cumulative occurrence of the primary composite endpoint was similar between the groups. Indeed, although a trend towards superiority was discernible, it had not reached a level of significance [72].

Additionally, it is noteworthy to acknowledge that a more intensive antithrombotic regimen (involving TAT, the use of prasugrel, and prolonged dual/triple therapy) results in higher bleeding risk. Conversely, a less aggressive antithrombotic approach is more advantageous in terms of diminished occurrences of bleeding events; nevertheless, the risk of the incidence of ischemic events will be higher.

In the PIONEER AF PCI, RE-DUAL AF PCI, AUGUSTUS, and ENTRUST AF PCI, the percentage of patients presenting with ACS, particularly those with ST-segment elevation myocardial infarction (STEMI), was limited. This subset of patients has an increased risk of ischemic events. Therefore, a more intensive antithrombotic approach is needed to determine the ideal duration of TAT.

Among 1875 patients on OACs from the Hungarian Myocardial Infarction Registry, no statistically significant disparities have been shown in terms of mortality, major adverse cardiovascular events (MACE), or transfusion rates between the OAC-treated group and propensity score (PS)-matched control group [73]. Furthermore, in PS-adjusted analyses within the OAC patients, the omission of aspirin therapy was associated with unfavorable outcomes [73].

It has been estimated that it is safer to include clopidogrel in the TAT than using ticagrelor or prasugrel [66] since newer P2Y12 receptor inhibitors have been associated with higher bleeding risk.

On the other hand, if we consider the clinical scenario of patients on DOACs performing an elective PCI, temporary discontinuation of the DOACs at least 24 h after the last intake has been suggested in order to allow a safe initiation of APT [18,74].

Additionally, it should be taken into account that different DOACs have demonstrated distinct cardiovascular risk profiles.

In the RE-LY trial, the administration of dabigatran 110 mg resulted in a similar rate of stroke and systemic embolism compared to patients on warfarin, alongside decreased occurrences of major bleeding. In contrast, patients treated with dabigatran 150 mg, in comparison to warfarin, have been shown to have a reduced rate of stroke and systemic embolism with comparable occurrences of major bleeding [40].

Importantly, the study indicated that patients undergoing anticoagulant therapy for AF remained vulnerable to myocardial infarction (MI), and there was an elevated risk of MI associated with dabigatran use. Significantly, both doses of dabigatran showed more MIs than warfarin, with an MI annual incidence of 0.53%, 0.72%, and 0.74% within the warfarin, dabigatran 110-mg, and dabigatran 150-mg groups, respectively. Nonetheless, a subsequent post hoc analysis exploring additional events involving stroke, bleeding, and MI resulted in revised findings that no longer indicated a statistically significant difference in the occurrence of myocardial infarction [75].

In a meta-analysis involving 196,761 patients from 28 RCTs aimed at assessing the CV long-term safety of DOAC treatment, Kupó et al. [76] identified significant differences in CV safety among oral anticoagulants. Treatment with rivaroxaban is associated with a reduced rate of MI.

A statistically significant reduction in the relative risk of MI has been revealed with rivaroxaban compared to both placebo and dabigatran.

These differences in the risk of MI may be considered in the development of personalized antithrombotic regimens, influencing the choice of treatment.

## 8. Pacemaker and Implantable Cardioverter-Defibrillator (ICD) Implantation

The safety of pacemaker or implantable cardioverter-defibrillator (ICD) implantation in anticoagulated patients has long been a major dilemma. However, in recent years, clinical studies showed that anticoagulation withdrawal and its continuation in the peri-surgery time resulted in similar rates of device pocket hematomas.

BRUISE CONTROL enrolled 659 patients undergoing pacemaker and ICD implantation. This study evaluated the strategy of continuing warfarin during surgery. The reduction in the development of pocket hematomas after the procedure has been associated with fewer subsequent infections [77].

The BRUISE CONTROL-2 study evaluated continuous versus discontinued DOAC strategies (dabigatran, rivaroxaban, or apixaban) in patients who underwent device implantation. For patients in the discontinuous arm, the last dose of rivaroxaban or apixaban was taken two days before surgery, while the timing of dabigatran discontinuation depended on GFR. The DOACs were restarted at least 24 h after the end of the procedure.

Data on 662 recipients who reported a low risk of developing significant hematoma with both strategies suggest that either interruption or continuation of the DOACs strategy is safe in the clinical scenario, at least for patients similar to those enrolled in the trial [78].

Recent ESC guidelines on cardiac pacing suggest that the choice of DOAC management strategy (continuous versus interrupted) is a matter of operator preference [79].

In addition, a recent meta-analysis, including the same BRUISE CONTROL-2 study, suggested that there are no differences in the occurrence of clinically significant pocket hematoma or thromboembolism in continuing versus stopping the DOACs. On the other hand, interruption may be the preferred strategy for most patients due to the ease of suspending and re-starting DOACs. A recent multicenter prospective study enrolled 789 patients with a high ischemic risk (median CHA2DS2-VASc score 4), of which 632 (80.1%) underwent pacemaker implantation. In this registry, DOACs were interrupted in 96% of patients for at least 12 h before the procedure and in 78% of patients of 12 h after the procedure; the rate of clinically relevant hematoma was 3.3%, and thromboembolic events occurred in 0.6% of the study population. The study highlighted that in the peri-procedural period, the bleeding risk prevails over the thromboembolic one, above all in patients with high ischemic risk [80].

Nonetheless, if continuous therapeutic anticoagulation is required, a continuation of DOACs should be preferred to bridge with LMWH [81].

## 9. Catheter Ablation of Atrial Fibrillation (CAAF)

Catheter Ablation of Atrial Fibrillation (CAAF) combines risks for both MB and thromboembolic events [82]. The most frequent major complications primarily pertain to the vascular access site [83]. Due to the potential periprocedural thromboembolic risk, the necessity for anticoagulation during the procedure is mandatory. Nonetheless, the presence of anticoagulation can complicate the management of bleeding complications [84].

The ultrasonography (US) application for femoral vein puncture in patients undergoing pulmonary vein isolation (PVI) resulted in a reduction in the incidence of both significant and minor vascular complications [85,86].

Importantly, administrating UFH before or right after the trans-septal puncture to obtain a stable target-activated clotting time of at least ≥300 s is required with the aim of minimizing the thrombotic risk [87].

An incidence of bleeding and thrombotic in-hospital complications of 1.9% and 0.2%, respectively, has been reported [88].

OAC treatment for at least three weeks before CAAF has been associated with reduced procedural thrombotic risk [87].

In recent decades, cardiologists have interrupted VKAs and adopted the bridging approach with LMWH before and after the procedure. Afterward, the COMPARE [89] trial demonstrated that patients who underwent CAAF continuing VKAs had a lower risk of periprocedural cerebrovascular accident (CVA) and minor bleeding (provided that INR remained in the therapeutic range) compared with those who experienced the VKAs interruption and LMWH bridging.

More recently, it has been shown that patients who underwent CAAF without interrupting DOACs did not develop more thrombotic and bleeding complications than those who experienced an uninterrupted VKAa regimen [90,91,92,93,94,95,96,97]. However, data from a metanalysis including 840 and 938 adults on uninterrupted and interrupted regimens, respectively, revealed that silent cerebral events occurred at a significantly higher frequency when anticoagulation was interrupted [98].

A significantly lower incidence of MB in patients with uninterrupted dabigatran compared to patients on uninterrupted warfarin without differences in terms of strokes or other thromboembolic events has been reported in the RE-CIRCUIT study [99].

Similarly, in VENTURE-AF, in patients who underwent CAAF on uninterrupted rivaroxaban, a lower incidence of hemorrhagic and thrombotic events has been shown compared to those on uninterrupted VKAs [96]. Also, the apixaban and edoxaban uninterrupted regimens have been shown to be safer and more effective than uninterrupted VKAs therapy in patients undergoing CAAF in AXAFA-AFNET 5 [94] and ELIMINATE-AF trials, respectively [100].

In the RYOUMA multicenter registry of 3.072 Japanese patients treated with CAAF, a low thromboembolic burden has been reported when DOACs were continued or minimally interrupted [101]. Periprocedural MB has been associated with several predictors such as female gender, long-standing AF, impaired renal and hepatic function, and intraprocedural administration of high heparin dose [101].

According to these findings, in patients undergoing CAAF, an uninterrupted DOAC strategy (un-DOACs) versus interrupted DOAC (in-DOACs) should be preferred [4,18,87]. The last dose of rivaroxaban and edoxaban should be given in the evening before the procedure, whereas apixaban and dabigatran must be administrated in the morning of the procedure in order to minimize the risk of thromboembolic and hemorrhagic events.

## 10. AF Patients Undergoing Non-Cardiac Surgery

It has been shown that there is a 25% likelihood that patients on DOACs will undergo a surgical or interventional procedure over a period of 24 months [102].

According to the most recent guidelines [102], if there is a need for surgery during the DOAC strategy, several factors, such as the kind of surgery and its specific bleeding risk, the patient’s characteristics, and the anticoagulation type, should be evaluated. Moreover, whether the procedure is urgent or not is crucial for patient management. The risk of bleeding increases significantly if manual compression is not possible. Patient-related factors include age, comorbidities, bleeding/thrombotic risk, and concomitant drugs [103].

In the presence of a high bleeding risk profile, any DOACs should be immediately discontinued in case emergency/urgent surgery should be adopted [18]. In these cases, it may be helpful to assess the anticoagulation effect prior to the surgery. Coagulation tests such as INR and aPTT are scarcely sensitive, although a normal aPTT during an emergency can help to rule out a relevant anticoagulant effect of dabigatran. A more accurate assessment of the anticoagulation effect for dabigatran may be obtained with the measurement of the diluted thrombin time (dTT), which has a linear correlation with the drug concentration and ecarin clotting time, while for apixaban, edoxaban, and rivaroxaban, DOAC-calibrated anti-factor Xa levels should be used although are not yet available in all laboratories [104].

Overall, it is advisable to assess the patient’s coagulation status with a comprehensive panel of clotting parameters and evaluate the need for reversal agents (idarucizumab, andexanet) and/or pro-hemostatic factors, including activated prothrombin complex concentrate (aPCC) and prothrombin complex concentrate (PCC) molecules.

The opportunity to postpone the intervention for 24–48 h should also be considered.

However, specific studies do not exist in this context for andexanet and PCC/aPCC. Its use should be considered when the conditions could endanger the life of the patient or the procedure.

The reversal agents might cause prothrombotic rebound, which requires interdisciplinary management regarding the early resumption of anticoagulant treatment [105,106,107].

In the RE-VERSE AD [108] and ANNEXA-4 trials [50], it has been shown that thrombotic events occurred in 4.8% and 10% of patients, respectively, within one month.

However, due to the lack of groups of control, it is extremely complex to conclusively establish whether thromboembolic events may be attributed to the intrinsic procoagulant antidote or to the hypercoagulability associated with patients‘ comorbidities and exacerbated by other factors such as inflammation, immobility, or blood transfusions [109].

Nevertheless, it is noteworthy that idarucizumab demonstrated no prothrombotic effect in experimental studies and among healthy volunteers. In contrast, andexanet exhibited a temporary elevation in D-dimer levels and other thrombin formation markers [110].

Until additional data become available, a black box warning for andexanet has been issued by the FDA [109]. However, the majority of thromboembolic episodes observed are typically manageable and are likely to be linked to discontinuing DOACs risk, particularly in cases of incomplete and delayed resumption of anticoagulation [111].

In this sense, in most cases, DOACs should be resumed promptly after a major bleeding episode and as soon as the thrombotic risk of thrombosis outweighs the rebleeding one. This approach is recommended, usually within one week [112].

However, in this context, a multidisciplinary approach should be adopted [111].

If surgery can be planned, a preliminary assessment of the bleeding risk is mandatory.

Procedures with minor bleeding risk should be performed at the minimum DOAC plasmatic levels (12–24 h after the last intake). However, when DOACs’ withdrawal is indicated, the discontinuation timing depends on patient characteristics (age, bleeding history, concomitant medications, renal function) and the type of DOACs. Assuming that bleeding risk factors are low and renal function is preserved, in adults on factor Xa inhibitors and dabigatran, DOACs should be interrupted 24 h before surgery.

While many of the minimally invasive procedures associated with a relatively low hemorrhagic risk can be carried out by temporarily interrupting treatment or without any interruption, conversely, in the case of high bleeding risk surgery, the last dose of DOACs should be taken at least 96 and 48 h before surgery for dabigatran and apixaban and for rivaroxaban and edoxaban, respectively.

In the PAUSE trial, with 3007 AF patients who underwent elective surgery and interrupted DOACs in the absence of heparin bridging, a lower incidence of arterial thromboembolism and MB was reported [113].

In a RE- LY sub-analysis that included AF patients receiving open-label dabigatran or warfarin therapy, those who experienced perioperative bridging had a higher rate of bleeding [114].

Based on these results, performing a heparin bridging strategy is currently not recommended. However, a bridging approach with UFH or low-dose dabigatran, in view of the rapid reversal of both drugs, can be taken in the case of a recent (<3 months) thromboembolic event or if the patient presented a thrombotic event during previous adequate DOACs interruption [114].

DOAC resumption after surgery depends on the patient’s hemostasis. DOACs can be restarted 6–8 h after the end of surgery in case of immediate and complete hemostasis. For low-risk and high-risk bleeding procedures, a resumption time of 24 h and 48–72 h has been proposed, respectively. When DOAC restart is postponed, postoperative thromboprophylaxis using a prophylactic LMWH dose can be considered 6–8 h after surgery.

If the oral administration of drugs is not indicated, the administration of heparin should be considered [113].

Cardiac surgery is considered a high-risk bleeding procedure. A standard DOAC interruption time of 48 h should be performed [115]. However, in patients at risk of DOAC accumulation, such as older patients or those with renal insufficiency, a longer interruption (>/=72 h) may be considered (Table 3).

The PAUSE study showed that, after 72 h of interruption, only a low percentage of patients had residual preprocedural DOAC levels ≥ 30 ng/mL [116], which is the cutoff value suggested by EACTS [115].

Surgery for a patient taking DOACs with an urgent heart condition should ideally be delayed [115].

However, the benefit associated with this postponement should be well-balanced between the delay in surgery and the risk of MB. Therefore, the timing between the last DOAC intake and the procedure should be monitored appropriately, and the remaining drug activity possibly assessed. If surgery cannot be postponed, idarucizumab for dabigatran and aPCC/PCC or andexanet for Xa inhibitors may be considered. It should be remembered that neither aPCC/PCC nor andexanet have been evaluated for this issue.

## 11. DOACs in Patients with Atrial Fibrillation and Malignancy

In AF patients with cancer, for thromboembolic prevention, ESC guidelines on AF should be used even if the CHA2DS2-VASc score and HAS-BLED score have not been extensively validated in patients with cancer [4]. An approach specific to anticoagulant therapy in cancer patients has been based on the acronyms T (thrombotic risk), B (bleeding risk), I (drug interactions), and P (patient access and preferences) [117].

Because of their drawbacks and the interactions among drugs in this setting, VKAs are rarely used in cancer patients; the effectiveness of LMWH in preventing stroke or systemic embolism (SE) in AF has not been well-assessed, and its use is only justified by its shown efficacy and safety in venous thromboembolism (VTE) [118].

In cancer patients, the use of DOACs for AF has not been examined in specific randomized controlled trials [119].

The absence of RCT, which specifically focuses on individuals with both cancer and AF, contributes to a state of uncertainty [120]. Thoughtfully designed clinical investigations are crucial for determining the most effective approach to anticoagulation for this population, according to their distinct thrombotic and bleeding vulnerabilities, potential DDI, and individual clinical attributes.

Post-hoc analyses of pivotal trials involving DOACS in AF, together with large observational studies suggest that the DOACs are safer and at least equally efficacious than VKAs in individuals with AF and active malignancy [121,122,123]. Patients with a previous diagnosis of cancer were poorly represented in the ROCKET AF trial (640 out of 14,264) [124]. In adults with and without a history of malignancy, rivaroxaban treatment has been shown to have similar efficacy and safety compared with warfarin. A history of malignancy increased the risk of bleeding and non-cardiovascular death, but not the risk of ischemic events [124]. Similarly, only 6.8% of patients in the ARISTOTLE trial had a history of cancer. MB, death, or stroke/SE were not significantly associated with a history of malignancy. Regardless of the cancer history, patients experienced similar benefits from apixaban compared to warfarin with regard to efficacy and safety [125]. In the ENGAGE AF-TIMI 48 study, 5.5% of patients had a new or recurring cancer diagnosed, with the most prevalent locations being the lung, prostate, and GI systems. Increased mortality and significant bleeding risk were linked to malignancy but not stroke or SE. The efficacy and safety profile of edoxaban compared to warfarin is preserved in AF patients who develop cancer, and it may be a more practical therapeutic option [123]. An extensive retrospective American database analysis in patients with underlying malignancy and AF showed that DOACs had a better safety profile than warfarin. Conversely, warfarin was linked to higher mortality as well as an increased risk of hemorrhagic stroke [126]. An administrative dataset was analyzed to verify if DOACs were effective and safe in AF patients with active cancer. Compared to warfarin users, DOAC users had lower or comparable rates of bleeding, stroke, and incident VTE [127]. According to data from subsequent retrospective Medicare and other commercial claims databases analysis on 40,271 AF adults with malignancy, apixaban was linked to a decreased risk of stroke/SE and MB compared to VKAs, whereas a comparable risk was reported in those on dabigatran and rivaroxaban [128]. Moreover, it has been recently pointed out that thromboembolic events and MB complications were significantly decreased in patients with AF and cancer on DOAcs when compared to those on VKAs [128].

A recent single-institution retrospective analysis including 1133 AF patients with malignancy, showed no differences in terms of CVA, GI bleeding, and intracranial hemorrhage (IH) when they were treated with DOACs instead of warfarin [122].

Data obtained from a Taiwan database between 2010 and 2017 indicated that among cancer patients with AF, DOAC use was correlated with a significant reduction in the occurrence of IS/SE, major bleeding, and ICH in comparison to warfarin [129].

Furthermore, in a recent comprehensive meta-analysis, including data from the ROCKET AF, ENGAGE AF-TIMI 48, and ARISTOTLE clinical trials, in addition to two additional investigations, it has been demonstrated that individuals with both cancer and AF treated with DOACS had a reduced or comparable occurrence of thromboembolic and hemorrhagic complications in comparison to those receiving warfarin treatment [130].

Moreover, a meta-analysis of randomized controlled trials, which compared DOACs with LMWH in AF patients with cancer, did not demonstrate a higher likelihood of major bleeding. Nonetheless, there was an increase in the risk of clinically significant non-major bleeding events, predominantly among patients diagnosed with gastrointestinal malignancies [131].

Evidence derived from the BLITZ-AF Cancer Registry, which enrolled 1514 individuals between 2019 and 2021 with both AF and cancer, indicates DOACs in the treatment of these specific patients [132].

There is evidence from the BLITZ-AF Cancer Registry on 1514 individuals (2019–2021) with AF and cancer suggesting that cardiologists encourage the adoption of DOACs in managing these patients [132].

No clear evidence is available on the choice of specific DOACs in this setting [119]. A retrospective database analysis from Taiwan reported that the use of dabigatran might be linked to a reduced risk of mortality from all causes and cancer-related causes when compared to rivaroxaban [133]. However, in recent decades, an increase in DOACS use in cancer patients has been observed, although a considerable proportion of them remain without anticoagulation [134]. Moreover, the use of DOACs in this subset of patients has been recently encouraged [118].

In individuals without substantial DDI and contraindications, DOACs should be taken into account for stroke prevention instead of LMWH and VKAs. The use of LMWH should only be considered in AF patients with malignancy, on the condition that they are not suitable for DOACs [135]. Nevertheless, active cancer patients represent a challenging population needing particular caution. The administration of oral anticoagulants in cancer patients may be complicated by other elements, such as DDI, renal impairment, and thrombocytopenia [136]. DDIs are not limited to anticancer agents, and supportive care drugs (i.e., antiemetics, opioids, etc.) must also be considered [137]. Given the rapidly changing clinical scenario, active cancer patients are likely to benefit from a closer follow-up strategy with frequent re-evaluations. Choosing the best anticoagulation plan for cancer patients requires a multidisciplinary treatment that considers personalized bleeding and thrombotic risks, DDIs, patient predilection, and routine clinical evaluation [136,138].

In conclusion, there is growing evidence supporting the safety and effectiveness of DOACs for stroke prevention in cancer patients with AF, making them a practical and patient-centered anticoagulation strategy.

## 12. DOACs in the Elderly Population

AF is a widespread arrhythmia in ≥75-year-old patients [14,139,140]. The elderly represent approximately 31–43% of the population in phase 3 DOAC trials, which shows that safety and efficacy were not affected by age [141]. The use of DOACs reduces the risk of IH more than VKAs [142] and, regarding extracranial bleeding, there is a significant interaction with age but only for dabigatran [143], which could be reduced by avoiding concurrent APT and using proton-pump inhibitors. Age can also be a direct or indirect (due to weight or kidney impairment) criterion for dose reduction, but it does not change the overall effect of DOACs. DOAC use in the elderly population is summarized in Table 4.

## 13. DOACSs and Frailty

Frailty must be carefully considered regarding the risk–benefit analysis of DOACSs because of the increased risk of renal impairment and falls. These conditions should not be a reason for choosing a no-anticoagulation strategy, but rather to choose the best drugs carefully with the adequate dose and to have regular follow-ups. As described by a recent meta-analysis, DOACs were preferred in patients at risk of falls because they were associated with fewer IH and ischemic strokes/SE than VKAs [144]. A higher benefit was shown with edoxaban and apixaban [145,146]. In particular, an 80% reduction in IH was observed with apixaban. Edoxaban was also an interesting option in light of the absolute reduction in MB compared with VKAs.

## 14. DOACs in Under and Overweight Patients

According to the World Health Organization, patients with a body mass index (BMI) < 18.5 kg/m^2^ are defined as underweight, whereas obese patients are those with a BMI > 30 kg/m^2^.

The use of fixed doses of drugs may result in lower exposure to the therapeutic agent in obese patients and increased exposure in underweight patients.

Current evidence for assessing the efficacy and safety of DOACs in obese patients derives from a subgroup analysis of clinical trials.

In the RELY study comparing the two different doses of dabigatran versus VKAs, 17% of patients with BMI > 36 kg/m^2^ were included, and there were no differences in terms of ictus or bleeding risk [147].

In the ENGAGE-AF TIMI 48 study evaluating edoxaban (30 and 60 mg) versus warfarin, in BMI categories between 18.5 and 40 mg/m^2^, no plasma variations were found, indicating comparable efficacy and safety [148,149].

According to recent evidence [150,151,152], the latest guideline of the International Society on Thrombosis and Haemostasis (ISTH) recommend the use of standard DOAC doses in patient ≤ 120 kg or a BMI ≤ 40 kg/m^2^ and avoid the use of DOACs in patients > 120 kg (BMI > 40 kg/m^2^) [147]. Accordingly, the recommendations of other more recent guidelines are similar [14,18,153], highlighting that in patients with grade II obesity (BMI 40–49 KG/m^2^), warfarin should be preferred, also considering the use of apixaban or edoxaban, whereas the use of dabigatran and rivaroxaban is not supported by sufficient studies [154].

However, new evidence suggested the use of DOACs between 35 and 150 kg despite limited pharmacokinetic data [155].

For severely obese patients (BMI > 50 kg/m^2^), data are not available for the use of DOACs, and only warfarin is indicated as an anticoagulant [18,155].

A retrospective analysis conducted on 348 individuals with AF and weight of ≥120 kg treated with DOACs indicated both safety and efficacy within the subset of patients. Nonetheless, it should be noted those with BMI > 50 kg/m^2^ were not adequately represented in the study, which suggests the necessity for further assessment in this specific population [156]. In light of these considerations, our knowledge of the effectiveness and safety of DOACs within this specific population remains a matter of uncertainty. Therefore, it is crucial that additional studies and trials are conducted to comprehensively address this issue.

Nevertheless, in severely obese patients, it has been postulated to consider the assessment of DOACs’ plasma levels [18,157]. Due to the higher reliability and the association with clinical outcomes, it is generally recommended to measure trough levels of the DOAC, although whether to evaluate the trough or peak plasma levels is currently a subject of ongoing research [18].

However, in light of the lack of data concerning the use of DOACs in the context of severe obesity, it becomes mandatory to conduct additional research to confirm the appropriateness of DOAC use in individuals with severe obesity. Furthermore, it is essential to investigate potential disparities and emerging patterns associated with the various DOACs in this particular population.

Below 35 kg, it is preferable to use warfarin, but DOACs may be used with particular attention to dose reduction and control of plasma concentrations.

In addition, other comorbidities such as older age, frailty, malignancy, and renal dysfunction, which are associated with low body weight, may increase the risk of stroke and bleeding. Furthermore, it should be considered that a reduced muscle mass, which often characterizes underweight patients, may result in an overestimation of renal function (especially if it is estimated with the MDRD formula [158].

In moderately and severely obese patients, the assessment of renal function with the CG formula might overestimate renal function. Therefore, it is advisable for these patients to use the MDRD equation and the Chronic Kidney Disease Epidemiology Collaboration (CKD-EPI) equation [159,160].

According to guidelines, a multidisciplinary approach should be adopted for patients with weight loss who need an anticoagulation strategy in order to avoid complications of AF treatment [4,161].

In conclusion, although it seems that indications for DOAC use may be extended to low- and high-weight patients, further large-scale studies are needed.

## 15. Adherence to Oral Anticoagulant Intake

The effectiveness and safety of pharmacologic interventions are affected by the patient’s ability to follow the recommended treatment regimen. Non-adherence rates to various cardiovascular (CV) drugs are considered to be at least 50% in the first treatment year, representing a significant burden to healthcare systems on a global scale [162]. This is also true for oral anticoagulants (OACs), which often require lifelong treatment. Many studies have explored the short- and long-term adherence rates of OAC among patients with AF or VTE in real-world settings [163]. The adherence rate in OAC users ranges widely depending on the setting and patient characteristics and decreases over time [164].

Several methods for estimating adherence are available. Medication refill adherence may be obtained through health administrative claim databases using the proportion of days covered (PDC) or medication possession ratio (MPR), and patients with PDC or MPR ≥ 0.80–0.90 are defined as adherent [165]. Self-reported adherence may be obtained through interviews with questionnaires. The most known are the Morisky Medication Adherence Scale 8-items (©MMAS-8) and the Medication Adherence Rating Scale (MARS-5) [166,167,168]. Other tools explore the quality of life or satisfaction with therapy in patients treated with OACs [167,168,169,170,171,172] (Table 5).

Finally, persistence is defined as continuous OAC use between the cohort entry date and the index date, and non-persistence is defined as patients who completely stopped their initial OAC treatment [173].

In the past, VKAs have been associated with suboptimal adherence and persistence due to various problems. In a regional claims database from China, among 33,463 patients with non-valvular (NV) AF who initialized warfarin, only 40.4% filled the first repeat prescription within three months [174].

Regarding DOACs, adherence in the real world may be lower than in clinical trials. A survey among 398 adults on DOACs reported a suboptimal adherence of 25% [175]. In other studies, the proportion of adherent DOAC users was 64.0% [176] and 90% [177]. In 474 patients prescribed dabigatran in 10 tertiary hospitals in South Korea, the adherence assessed by the PDC was 93.5 ± 5.5% at one month and 96.4 ± 8.4% at six months among the persistent patients [178].

Among 21,028 Swedish AF patients prescribed with DOACs between 2011 and 2018, 90% adherence has been reported [179]. In Kaiser Permanente Southern California, in 18,920 AF patients who initiated DOACs between 2012 and 2018, long-term (3.5 years) adherence rates of 85.2%, 10.5%, and 4.2% for adherence, interruption within six months, and gradual discontinuation therapy have been reported [180].

Some studies have focused on patients switching from VKAs to DOACs. In the Netherlands, among 1399 patients on DOACs previously treated with VKAs, persistence and adherence rates of 94% and 86%, respectively, have been shown [181]. The Switching Study analyzed British patients anticoagulated for stroke prevention in AF or secondary prevention of VTE who switched from warfarin to DOACs. After the first year, 39% and 23% of sub-optimal adherence and non-adherence were reported, respectively [182].

Many studies considered adherence to both VKAs and DOACs together. An analysis of 837 AF adults ≥ 75 years on OACs ≥ 3 months observed poor adherence in 27.9% of patients [183]. In a cross-sectional, single-center observational survey study, adequate treatment adherence was found in three-quarters of patients [184]. Among patients performing elective DC-cardioversion at Haukeland University Hospital, 89% were compliant with the prescribed OACs [185]. In 306 participants followed in specialized AF clinics in Canada, the mean self-reported adherence on the ©MMAS-8 was 7.28/8 [186]. In a study of 33,311 patients using OACs within 12 months after hospital discharge, approximately 75% were considered “adherent”, with a mean PDC of 95.6–98.1% [187]. Furthermore, there is heterogeneity among non-adherent patients regarding the rate and timing of the decline in their medication, as has been confirmed by data from 19,749 patients in British Columbia and Canada (1996–2019), with different adherence profiles, which varied from consistent adherence (74%), “early non-adherence” (12%), and “early non-adherence and partial recovery” (10%) to “gradual non-adherence” (4%) [188].

## 16. Differences between VKAs and DOACs

Some studies compared adherence and persistence between VKAs and DOACs, as there was initially a suspicion that DOAC adherence may be poor due to the absence of anticoagulation laboratory monitoring [164]. In a retrospective study conducted among 317 AF patients enrolled in the Medicare Advantage Plan from 2016 to 2019, DOAC adherence gradually and rapidly declined in 40.4% and 20.8% of patients, respectively, whereas 38.8% of adherence was observed. In contrast, in patients on warfarin, adherence gradually diminished in 8.9%, while 21.7% occasionally discontinued therapy; finally, 59.4% of them regularly assumed VKAs [164].

Primary nonadherence to OACs of 5.62% has been reported among 18,715 Spanish patients with AF [189]. Based on the Dutch Foundation of Pharmaceutical Statistics (2012–2016), non-persistence to DOACs was 34% at one and 64% at four years, compared to 22% and 36% at four years for VKAs [190].

Other studies, however, demonstrated better adherence and persistence with DOACs. Specifically, 77% persistence in patients with apixaban versus 53% in those with VKAs has been shown [187]. Among 26,029 AF Israelite patients, medication adherence was 88.9%, 84.9%, 83.6%, and 55.8% for rivaroxaban, apixaban, dabigatran, and warfarin, respectively [191]. Among 122,870 Hungarian patients, the one-year persistence of DOACs was 65.7%, while that of VKAs was 39.0% [192]. Among 7013 patients from the CODE-AF Korean registry, persistence over six months declined to 88.3% and 95.5% for VKAs and DOACs, respectively [193].

Data from a survey conducted on 765 patients with AF revealed that practical issues with medication intake occurred more frequently in VKAs than in DOAC users [194]. In the UK primary care Health Information Network (2011–2016), in 36,652 individuals with AF, adherence was 55.2%, 51.2%, 66.5%, 63.1%, and 64.7% and one-year persistence was 65.9%, 63.4%, 61.4%, 72.3%, and 78.7% for all OACs, VKAs, dabigatran, rivaroxaban, and apixaban, respectively [195]. Overall, the percentage of non-adherent and non-persistent patients, regardless of liver disease, was higher in warfarin patients compared with those receiving apixaban and rivaroxaban [43]. In a meta-analysis of 395,593 adults, DOAC use resulted in less non-persistence compared to VKAs: Apixaban, 0.33; rivaroxaban, 0.47 (0.36, 0.61); and dabigatran, 0.61 (0.44, 0.85) [43].

Finally, other studies showed that drug class does not seem to impact adherence significantly [169,171,186].

According to the results from a database of cancer patients who received diagnoses between 2009 and 2015, those on DOACs had a higher persistence compared with LMWH (median 116 versus 34 days). With adherence defined as PDC ≥ 80%, no significant difference occurred (95.6% with DOACs versus 94.6% with LMWH, *p* = 0.33). Adherence, which is considered to be PDC ≥ 95%, has been reported in 73% and 81% of patients on DOACs and LMWH, respectively [196].

## 17. Differences in Adherence between Single DOACs

Some studies showed greater adherence and persistence with rivaroxaban and edoxaban, likely due to their once-a-day administration. In a meta-analysis evaluating 80,230 patients with AF in a U.S. real-world setting, patients on rivaroxaban were more adherent than those on dabigatran [197]. In a retrospective evaluation of AF patients in Japan, drug persistence at three years was 69%, 57%, and 67% with rivaroxaban, dabigatran, and apixaban, respectively [177]. In a therapy-naïve group, the one-year persistence with rivaroxaban was 65.7%, with apixaban was 62.6%, and with dabigatran was 59.2% (*p* < 0.01) [192].

In a study on 2932 patients treated with dabigatran, the likelihood of maintaining treatment continuity after 24 months was approximated at 70%. Noteworthy trends emerged, indicating that patients from North America and those with paroxysmal, persistent, or symptomatic AF were more likely to discontinue dabigatran therapy [198].

Indeed, considering the growing prevalence of AF in the elderly, advancing age, cognitive decline, multimorbidity, and concurrent polypharmacy may potentially contribute to a relatively diminished adherence, particularly in relation to dabigatran and apixaban due to twice-daily dosing regimens.

Other studies, however, give contrasting results.

The potential factors contributing to disparities in adherence among different DOACs might include their respective side effect profiles, marketing strategies, and variations in procurement and prescription practices, patient or physician preferences, or other unexplored variables.

Furthermore, the patient’s viewpoint has been recognized to play a pivotal role in anticoagulant selection [164]. These preferences may potentially influence treatment adherence and the decision to transition to an alternative OAC.

Finally, other studies did not observe significant differences in adherence or persistence among single DOACs. Over a 5.5-year study period, the overall PDC was 0.71 ± 0.21, without any significant difference between dabigatran, rivaroxaban, and apixaban [184].

## 18. Patient Characteristics Associated with Adherence and Persistence to OACs

It has been widely acknowledged that adherence and persistence are affected by physician-and healthcare system-related determinants. The most common causes for non-adherence are forgetfulness, a busy lifestyle or complexity, and changes in the therapeutic schedule.

## 19. Age

In 192 participants at a university-affiliated hospital in Taiwan, medication adherence correlated significantly with age (*p* < 0.05) [199]. Multivariable analyses revealed that age ≤ 60 years is a predictor of non-adherence and non-persistence [190], whereas advanced age is associated with higher adherence rates [181,186,187,191,200]. However, higher scores in fragility scales in the elderly are associated with poorer adherence [183], and in 785 consecutive outpatients, those over 75 years were more likely to forget the assumption of OAC [201].

## 20. Gender

Most studies report that females were more likely to be adherent compared to males [187,201], with an OR of 1.69 (95% CI, 1.08–2.64; *p* 0.023) [200,202]. However, in multivariable analyses, the female sex is a predictor for DOACs non-persistence [190].

## 21. Socioeconomic Status and Education

Socioeconomic status, income, and educational levels do not seem to be associated with medication adherence [191,203]. However, in the nationwide registry-based FinACAF cohort, covering 74,222 adults with AF on DOACs, higher income or education has been associated with adherence to DOACs (OR 1.18 (1.12–1.25) and 1.21 (1.15–1.27), respectively) [165].

## 22. Comorbidities

Diabetes, valve disease, a prior history of cancer, and the absence of hypertension are significantly associated with OAC discontinuation [177,193]. Having dementia and liver dysfunction [43] are associated with non-adherence (due to several causes such as forgetting, schedule disruptions, side-effects, economic reasons, and the so-called “symptom management” when patients decide to self-regulate the intake according to their symptoms [180]. In a multivariate analysis, gastrointestinal (GI) discomfort was the only predictor of dabigatran discontinuation and low adherence [178]. VTE patients prioritize OAC over other therapies, whereas AF patients do not [204]. In contrast, better adherence to OACs has been reported in patients with lower low-density lipoprotein cholesterol (LDL-C) and glucose levels [191] and in those at a greater risk of stroke [187,205,206]. Nevertheless, patients with lower CHA2DS2-VASc were more likely to be non-adherent [180]. An increasing Charlson Co-morbidity Index score, which predicts mortality risk, was linked to a lower risk of non-adherence and non-persistence for all OACs [43,195,205,206].

## 23. Previous Thrombosis and Bleeding

Having experienced a thrombotic episode seems to be associated with better adherence [201]. Conversely, a history of minor bleeding on DOACs could be a predictor of nonadherence (OR, 1.9; 95% CI, 1.3–2.8) [181], although according to other studies, it only marginally influenced treatment adherence [166]. Notably, an increased risk of bleeding results in better adherence [205,206].

## 24. Psychological Factors

It has been generally accepted that greater satisfaction with medications results in higher adherence [186]. In the ROCKET AF population, satisfaction was similar in rivaroxaban and warfarin patients [170]. Nevertheless, other studies confirmed no significant difference in quality of life but a significantly greater satisfaction with DOACs compared to warfarin (*p*, 0.001) [171]. Treatment satisfaction and concerns about making mistakes when taking OACs are significant predictors of adherence [184,200].

Greater concerns at baseline are significantly associated with anticoagulant nonadherence [204], but depression and drug attitudes are not significantly related to anticoagulant adherence [207]. Perceived barriers and self-efficacy significantly influenced OAC adherence [169].

## 25. Polypharmacy

Polypharmacy is inversely associated with primary nonadherence [189,199]. However, treatment with chronic cardiovascular disease drugs is related to high adherence [187], and multivariable analyses revealed that no concomitant drug use was a predictor for the non-adherence and non-persistence of DOACs [190].

## 26. Frequency of Administration

A twice-daily dosing regimen is a predictor of nonadherence (OR 1.9; 95% CI 1.3–2.6) [181,201].

The twice-daily administration may be an independent factor in older people, causing inadequate adherence [208].

## 27. Factors Related to the Health System

An exploratory study among general physicians practicing in Western Australia showed that access to a cardiologist could impact adherence [209], whereas the lack of specialistic evaluation was a predictor of nonadherence (OR 1.6; 95% CI 1.1–2.2) [181]. Electronic transmission of prescriptions was inversely associated with primary nonadherence [189]. Scarcely involving patients in the decision of anticoagulation and unclear information have been considered causes of non-adherence [175].

OAC adherence and persistence are also associated with lower costs, and what is more, the cost of medication is widely regarded as a significant predictor of adherence [200].

Indeed, it has been shown that among 17,558 patients with AF who initiated DOACs from 2012 to 2018 and were covered by health insurance, those who afforded higher costs were more likely to be adherent compared to those who paid less [210]. Moreover, the total medical costs of adherent patients have been shown to be significantly lower than those of nonadherent patients [168,206].

## 28. Consequences of Non-Adherence and Non-Persistence

Improving OAC adherence in AF patients should be a clinical priority since data indicate that adherent patients are more likely to have better outcomes [211].

A short duration in the therapeutic range (TTR) was associated with an irregular assumption of VKAs [201].

Patients with a suboptimal TTR have been shown to have a higher stroke risk [179]. Suboptimal adherence (>5% missed doses) resulted in higher thromboembolic complication occurrence [212]. In a study on AF patients admitted to seven tertiary Lebanese hospitals, high medication adherence resulted in a lower risk of stroke [213]. In contrast, early discontinuation of DOACs leads to a higher risk of SE, especially 12 months after DOAC initiation [180]. The adjusted OR for the risk of cerebral infarction for low-adherence patients (<80% adherence rate) versus. high-adherence patients (100% adherence rate) was 9.69 (95% ci 3.86–24.3; *p* < 0.001) [102]. In the Korean National Health Insurance Service database, which included 96,197 patients with AF who initiated OACs in 2013-16, adherent DOAC users versus non-adherent users were at lower risk of ischemic stroke/SE and acute myocardial infarction (AMI). In contrast, there was no significant risk alteration for MB. Notably, in patients on DOACs, the adverse outcomes rate was associated with poor adherence [176]. Data from Dutch, Italian, and German databases confirmed that lower adherence results in thromboembolic risk [173]. Conversely, lower severity incidence has been reported in those with adequate DOACs [214]. According to Japan Medical Data, OAC administration in patients with an MPR ≥ 90% was significantly associated with a lower risk of dementia [215].

## 29. How to Improve OAC Adherence and Persistence in Patients

Starting anticoagulation is not enough; we must ensure that patients remain adherent for life. Factors that can be modified may be used to improve therapy adherence.

First, patient motivation should be investigated when beginning an OAC (through patient-modifiable health beliefs) and during follow-up. Where there is suspected or declared non-adherence, strategies must be developed together with the patients to improve adherence [216].

The physician–patient relationship is crucial. There remains a substantial unmet need to educate patients with AF or VTE, focusing on good knowledge and the correct perception of OAC advantages and disadvantages. Adherence counseling should be systematically and repeatedly implemented. Moreover, a team-based evaluation of patients may improve adherence. However, Noseworthy and colleagues enrolled 922 evaluable patients in SDM and usual care (UC) without finding differences between arms in either primary (78% of patients in the SDM arm filled their first prescription versus 81% in the UC arm) or secondary adherence to anticoagulation [217]. Frequent consultation with a medical specialist should be encouraged.

Moreover, in the Eastern Health Adult Outpatient Thrombosis Service in Canada, it has been demonstrated that other professional categories, including nurses, are essential in promoting adherence [218]. The role of tools may be helpful. A simple calendar reminder of drug intake effectively improves OAC therapeutic adherence [219]. Adherence devices that use digital technologies (Smartphone and tablet applications, smart pill bottles) could help improve patient compliance by providing them with timely information through push notifications [220]. They have shown to be successful in several clinical trials but are not used broadly in clinical practice [221]. Technological tools such as smartphones or tablets are thought to improve adherence. However, all available technologies for monitoring medication adherence also have several drawbacks. Electronic pill boxes, bags, and bottles rely on occurrences of container openings as indicators of medication consumption. However, instances of pocket dosing and curiosity-driven openings may influence the accuracy of the devices in estimating patient adherence [222,223,224,225].

## 30. Conclusions

Nowadays, DOACs are considered the first-line strategy for stroke prevention in AF.

A more beneficial profile in terms of efficacy and safety has been reported in AF patients compared to warfarin [226]. Significantly, a lower incidence of ischemic and severe bleeding complications has been reported in patients treated with the standard-dose DOACs adjusted for renal function compared with patients on warfarin. Moreover, it has been established that the use of lower doses, if not based on renal functionality assessment of DOACs, has not been associated with a reduced risk of stroke compared to warfarin and that standard-dose use should be supported in the absence of the specific conditions that require lower doses of DOACs [227].

It is mandatory to consider the half-life of DOACs in order to establish whether to interrupt DOACs before elective procedures and how the interruption period should last, distinguishing the procedures associated with a low bleeding risk from those with a moderate and high risk of bleeding and adopting a more appropriate strategy according to this risk. Moreover, renal function must be taken into account for DOACs, considering moderate renal impairment on dabigatran, and all patients with severe renal dysfunction require specific considerations. Moreover, DDI should be carefully considered, with particular attention to the strong inducers or inhibitors of P-gp drugs due to the fact that these interactions may imply serious adverse events. Another critical issue concerns those patients on a long-term DOAC who require nonemergent invasive coronary procedures. In these cases, what worries the physician most is the management of antithrombotic therapy due to the fact that they deal with the decision of interrupting for a period before performing the coronary invasive procedure or whether it could be better not to interrupt OAC [197].

Both these approaches may be adopted in clinical practice, taking into account the patient’s bleeding and thrombotic risk characteristics and the clinical context in which the procedure is performed. However, an uninterrupted OAC strategy may be adopted and considered in patients at high thrombotic risk, mainly if they are on VKAs, and in patients with NSTE-ACS requiring a nonemergent PCI for NSTE-ACS, considering the fact that the interrupting OAC may be associated with the delayed time of treatment and lengthening of hospitalization. Notably, the uninterrupted strategy should be reserved for those patients who are neither at high bleeding risk nor at increased risk of vascular complications or the poor possibility of using radial access [228].

Another critical issue is the DOACs’ effectiveness in obese and underweight patients.

An ambulatory assessment of anti-Xa level for obese adults and a team-based evaluation involving hematologists and, additionally, drug-level monitoring for underweight patients have been proposed as possible solutions [229]. Nevertheless, DOACs seem to be more effective and safer than OACs among all BMI patients [230].

Finally, several concerns exist about the safety and efficacy of DOACs in patients with malignancies due to the increased bleeding risk and the prothrombotic state, which typically characterizes the malignancy state and anticancer therapies and potential DII and altered pharmacokinetics.

Although a great deal of real-world evidence supports the use of DOACs in cancer patients with AF [128], an accurate and tailored evaluation including consideration of where the cancer is localized, the grade of disease, the general status, and concomitant therapies are needed for these patients.

Additionally, it is noteworthy that, due to the concurrent presence of other factors that frequently coexist with AF, the anticoagulation strategy does not completely prevent the occurrence of ischemic stroke among such patients. Indeed, it has been recently highlighted that DOACs, which act on the embolic factor associated with AF, might be inadequate in addressing other concurrent thrombotic, proatherogenic, and proinflammatory elements linked to the pathogenesis of both stroke and AF, resulting in a non-comprehensive protection against the incidence of ischemic stroke in AF patients [231,232].

In conclusion, many unresolved unexplored issues remain for DOACs. Further research on DOACs in specific clinical contexts is needed in order to improve their use in clinical practice.

## Figures and Tables

**Table 1:** Drug–Drug Interactions (DDIs) of DOACs.

Drugs	Strong	Moderate to Weak
Pgp or combined CYP3A4/5/Pgp inhibitors	Clarithromycin, Cobicistat, Ketoconazole, Itraconazole, Dronedarone, Erythromycin, Posaconazole, Ritonavir, Voriconazole	Amiodarone, Cyclosporine, Diltiazem, Ticagrelor, Verapamil, Quinidine
CYP3A4/5 inhibitors	Boceprevir, Grapefruit Juice	Fluconazole
P-gp inducers	Rifampin	
CYP3A4/5 inducers	Phenytoin	
CYP3A4/5 inducer+combined P-gp inducer	Apalutamide, Bosentan, Carbamazepine, Phenobarbital, St. John’s Wort	

Pgp: P-glycoprotein; CYP3A4/5: cytochrome P450 3A4/5.

**Table 2:** Dose for each DOAC according to GFR.

Dose for Each DOAC According to GFR				
GFR (mL/min)	Dabigatran	Rivaroban	Apixaban	Edoxaban
>50	150/110 mg BID	20 mg OD	2.5/5 mg BID	60 mg OD
50–30	150/110 mg BID	15 mg OD	2.5/5 mg BID	30 mg OD
30–15	Contraindicated	15 mg OD	2.5 mg BID	30 mg OD
<15 or dialysis	Contraindicated	Contraindicated	Contraindicated	Contraindicated

GFR = Glomerular Filtration Rate.

**Table 3:** DOACs interruption according to renal function.

DOAC		CrCl				
		≥80 mL/min	50–79 mL/min	30–49 mL/min	15–29 mL/min	<15 mL/min
Dabigatran	Low BR	≥24 h	≥36 h	≥48 h		
	High BR	≥48 h	≥72 h	≥96 h		
Apixaban Edoxaban Rivaroxaban	Low BR	≥24 h			≥36 h	
	High BR	≥48 h				

Abbr: DOAC: Direct oral anticoagulants; CrCl Creatinine Clearance; h: hours; mL/min: milliliters per minute; BR: Bleeding Risk. Legend: 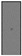 Contraindicated.

**Table 4:** DOACs in elderly.

	Study	≥75 Years	Overall Relative Risk vs. VKAs for Stroke/SE, RR (95% CI)	Overall Relative Risk vs. VKAs for Primary Safety, RR (95% CI)
Dabigatran	RE-LY [40]	7258 (40%)	110 mg, 0.91 (0.74–1.11) 150 mg, 0.66 (0.53–0.82)	110 mg, 0.80 (0.69–0.93) 150 mg, 0.93 (0.81–1.07)
Rivaroxaban	ROCKET-AF [42]	6229 (44%)	0.88 (0.75–1.03)	1.03 (0.96–1.11)
Apixaban	ARISTOTLE [41]	5678 (31%)	0.79 (0.66–0.95)	0.69 (0.60–0.80)
Edoxaban	ENGAGE-AF [21]	5668 (40%)	0.87 (0.73–1.04)	0.80 (0.71–0.91)

Abbr: VKAs: Vitamin K Antagonist; mg: milligram; Stroke/systemic embolism: SE; RR: relative risk; CI: confidence interval.

**Table 5:** Tools exploring the quality of life or satisfaction with therapy in patients treated with OACs.

Tools for Evaluating Quality of Life or Satisfaction in Anticoagulated Patients
AF-related symptom subscale of the AF Severity Scale
Knowledge of Warfarin Anticoagulation Treatment Scale
Satisfaction Scale about Service and Warfarin Treatment
Perceived benefits subscale of the Beliefs about Anticoagulation Survey
Concerns about Anticoagulation Therapy Scale
Self-efficacy for Appropriate Medication Use Scale
Short-form Adherence to Refills and Medications Scale
Short-form Adherence to Refills and Medications Scale
Perception of Anticoagulant Treatment Questionnaire
Anti-Clot Treatment Scale
Treatment Satisfaction Questionnaire for Medication version II

## Data Availability

Not applicable.
